# PICT1 expression is a poor prognostic factor in non-small cell lung cancer

**DOI:** 10.18632/oncoscience.43

**Published:** 2014-05-25

**Authors:** Kyoko Okamura, Koichi Takayama, Kohichi Kawahara, Taishi Harada, Miki Nishio, Kohei Otsubo, Kayo Ijichi, Mikihiro Kohno, Eiji Iwama, Akiko Fujii, Keiichi Ota, Takaomi Koga, Tatsuro Okamoto, Akira Suzuki, Yoichi Nakanishi

**Affiliations:** ^1^ Research Institute for Diseases of the Chest, Graduate School of Medical Sciences, Kyushu University, Japan.; ^2^ Department of Molecular Oncology, Graduate School of Medical and Dental Science, Kagoshima University, Japan.; ^3^ Division of Cancer Genetics, Medical Institute of Bioregulation, Kyushu University, Japan.; ^4^ Division of Pathophysiological and Experimental Pathology, Department of Pathology, Graduate School of Medical Sciences, Kyushu University, Japan.; ^5^ Department of Surgery and Science, Graduate School of Medical Sciences, Kyushu University, Japan.; ^6^ Faculty of Medical Sciences, Department of Comprehensive Clinical Oncology, Kyushu University, Japan.

**Keywords:** PICT1, TP53, lung cancer, lymphatic invasion, GLTSCR2

## Abstract

PICT1 is a key regulator of the MDM2–TP53 pathway. High mRNA expression levels of PICT1 are associated with poor prognosis in several cancers with wild-type *TP53*. In this study, we identified the PICT1 protein expression profile in non-small cell lung cancer (NSCLC) with wild-type *TP53* in the nucleolus and cytoplasm, and revealed the relationship between PICT1 expression and patient clinicopathological factors. PICT1 expression in the tumor cells of 96 NSCLC patients with wild-type *TP53* was evaluated by immunohistochemistry. Forty-three of 96 (44.8%) NSCLC samples were positive for nucleolar PICT1, while 40/96 (41.7%) NSCLC samples were positive for cytoplasmic PICT1. There was no correlation between nucleolar PICT1 expression and clinicopathological factors. However, cytoplasmic PICT1 expression was significantly correlated with sex, smoking history, differentiation, lymphatic invasion and pathological stage. In multivariate analysis, lymphatic invasion was significantly associated with cytoplasmic PICT1 expression (hazard ratio: 5.02, *P* = 0.026). We scrutinized PICT1 expression in samples of NSCLC with wild-type *TP53*, and showed a correlation between cytoplasmic PICT1 expression and several clinicopathological factors in these patients. Our results indicate that cytoplasmic PICT1 expression is a poor prognostic factor and is associated with tumor progression via lymphatic invasion in these patients.

## INTRODUCTION

Lung cancer is a common cause of cancer-related death worldwide. Patients with early-stage lung cancer who undergo curative surgical resection often die from recurrent disease or distant metastases. The long-term survival rate for lung cancer patients remains low [1]. Thus, personalized and targeted lung cancer therapy dependent on the characteristics of each patient is urgently required.

TP53 activation by ribosomal biogenesis stress is important for tumor suppression [2]. Protein interacting with carboxylterminus-1 (*PICT1*) gene, also called glioma tumor suppressor candidate region gene 2 (*GLTSCR2*), is located at human chromosome 19q13.32, which is frequently altered in human tumors [3]. We previously identified that PICT1 is an important regulator that acts primarily through ribosomal protein 11 (RPL11) and murine double minute 2 (MDM2) to inhibit TP53 responses against nucleolar stress [4, 5]. PICT1 binds to RPL11 in the nucleolus and prevents it from interacting with MDM2, thus blocking TP53 accumulation and activation. We also showed that low PICT1 expression was associated with better prognosis in colorectal tumors, esophageal tumors, hepatocellular carcinomas and gastric cancers with wild-type *TP53* [4, 6, 7]. Thus, PICT1 is a useful prognostic marker for these cancers [4]. However, PICT1 has been considered as a suppressor of tumor development [8-10]. Overexpression of GLTSCR2 induced phosphatase and tensin homolog (PTEN)-dependent apoptotic cell death in glioblastoma cells [10]. Knockdown of PICT1 promoted cell proliferation and anti-apoptosis [11]. Low PICT1 expression in astrocytic glial tumors and ovarian cancers was correlated with high malignant progression [8, 9, 12]. There are no reports available concerning PICT1 expression in lung cancer, and its relationship with clinical factors in this cancer type is unknown.

In this study, we investigated the expression of PICT1 in surgically resected non-small cell lung cancers (NSCLC) without *TP53* mutation using immunohistochemistry. We identified a relationship between PICT1 expression and clinicopathological variables in NSCLC with wild-type *TP53*.

## RESULTS

### PICT1 expression in lung cancer tissue

The patients' characteristics are summarized in Table 1. Clinicopathological factors, including age, sex, smoking history, differentiation, vascular invasion, lymphatic invasion, pleural invasion, T status, N status, pathological stage, epidermal growth factor receptor (EGFR) mutation status (41 patients with sensitive mutations, 49 patients without mutations, six patients with unknown mutational status) and histological subtype, were evaluated. There were 53 males and 43 females, with a mean age of 67.7 ± 9.3 years.

**Table 1:** Correlation between PICT1 expression and clinicopathological factors in 96 non-small cell lung cancer samples (wild-type *TP53*)

Factors		nucleolus				cytoplasm		
		Total patients	PICT1-positive	PICT1-negative	*P* value	PICT1-positive	PICT1-negative	*P* value
		n = 96	n = 43	n = 53		n = 40	n = 56	
			(%)	(%)		(%)	(%)	
Age	< 70	55	24 (44)	31 (56)	0.792	25 (45)	30 (55)	0.382
	≥ 70	41	19 (46)	22 (54)		15 (37)	26 (63)	
Sex	Male	53	23 (43)	30 (57)	0.760	28 (53)	25 (47)	0.013*
	Female	43	20 (47)	23 (53)		12 (28)	31 (72)	
Smoking history	Smoker	56	26 (46)	30 (54)	0.703	30 (54)	26 (46)	0.005*
	Never smoked	40	17 (42)	23 (58)		10 (25)	30 (75)	
Differentiation	Well	46	20 (43)	26 (57)	0.804	11 (24)	35 (76)	0.001*
	Moderately + poorly	50	23 (46)	27 (54)		29 (58)	21 (42)	
Vascular invasion	Absent	71	34 (48)	37 (52)	0.301	26 (37)	45 (63)	0.093
	Present	25	9 (36)	16 (64)		14 (56)	11 (44)	
Lymphatic invasion	Absent	84	38 (45)	46 (55)	0.816	31 (37)	53 (63)	0.012*
	Present	12	5 (42)	7 (58)		9 (75)	3 (25)	
Pleural invasion	Absent (P0)	82	38 (46)	44 (54)	0.456	31 (38)	51 (62)	0.065
	Present (P1-3)	14	5 (36)	9 (64)		9 (64)	5 (36)	
T status	T1	62	26 (42)	36 (58)	0.216	22 (35)	40 (65)	0.240
	T2	28	16 (57)	12 (43)		14 (50)	14 (50)	
	T3	5	1 (20)	4 (80)		3 (60)	2 (40)	
	T4	1	0 (0)	1 (100)		1 (100)	0 (0)	
N status	N0	86	40 (47)	46 (53)	0.577	33 (38)	53 (62)	0.085
	N1	6	2 (33)	4 (67)		5 (83)	1 (17)	
	N2	4	1 (25)	3 (75)		2 (50)	2 (50)	
Pathological stage	I+II	89	41 (46)	48 (54)	0.361	34 (38)	55 (62)	0.012*
	III+IV	7	2 (29)	5 (71)		6 (86)	1 (14)	
EGFR mutation	No mutation	49	19 (39)	30 (61)	0.237	24 (49)	25 (51)	0.096
	With mutation	41	21 (51)	20 (49)		13 (32)	28 (68)	
	unknown	6	3 (50)	3 (50)		3 (50)	3 (50)	
Histological	Adenocarcinoma	86	37 (43)	49 (57)	0.308	33 (38)	53 (62)	0.056
subtype	Squamous cell carcinoma	10	6 (60)	4 (40)		7 (70)	3 (30)	

* *P* < 0.05

**Figure 1:**
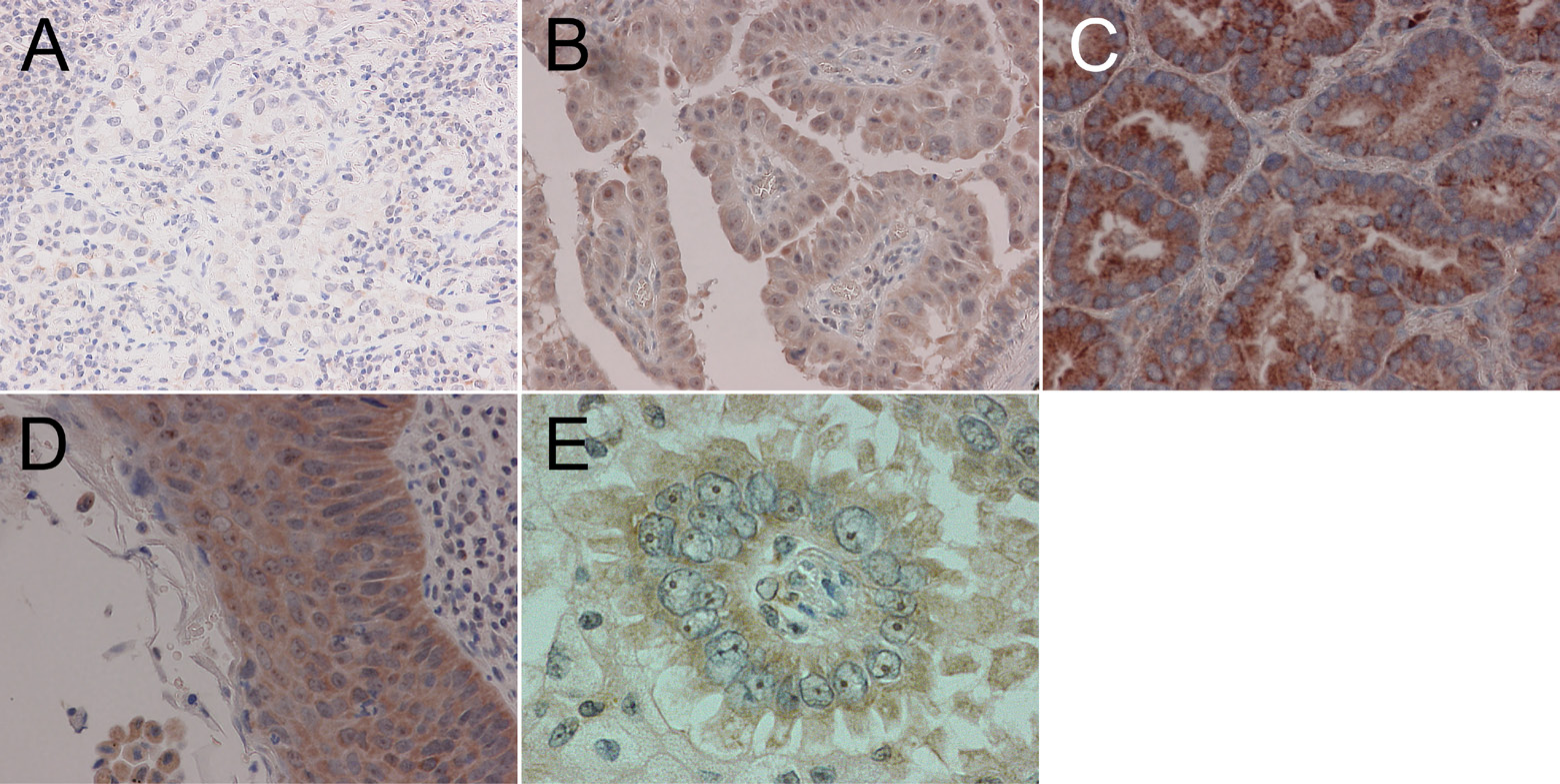
Nucleolar and cytoplasmic PICT1 expression in non-small cell lung cancer (A) Negative nucleolar and cytoplasmic PICT1 staining in adenocarcinoma (magnification 200×). (B) Positive nucleolar and cytoplasmic PICT1 staining in adenocarcinoma (200×). (C) Negative nucleolar and positive cytoplasmic PICT1 staining in adenocarcinoma (200×). (D) Positive nucleolar and cytoplasmic PICT1 staining in squamous cell carcinoma (200×). (E) Positive nucleolar PICT1 staining in adenocarcinoma (1200×).

Representative images of immunohistochemical staining of PICT1 expression in the nucleolus or cytoplasm are shown in Figure 1. Nucleolar or cytoplasmic staining was evaluated independently. Forty-three of 96 (44.8%) NSCLC samples were positive for PICT1 in the nucleolus. Forty of 96 (41.7%) NSCLC samples were positive for cytoplasmic PICT1. Seventeen patients were positive for both nucleolar and cytoplasmic PICT1, while 26 patients were positive in the nucleolus only, 23 patients in only the cytoplasm, and 30 patients were negative in both the nucleolus and cytoplasm (Table 2). There was no significant correlation between nucleolar and cytoplasmic PICT1 expression using chi-square tests (*P* = 0.703).

**Table 2:** Relationship between nucleolar and cytoplasmic PICT1 expression

	cytoplasm PICT1-positive (n=40)	cytoplasm PICT1-negative (n=56)	*P* value
nucleolus PICT1-positive (n=43)	17	26	0.703
nucleolus PICT1-negative (n=53)	23	30	

### Correlations between PICT1 expression and clinicopathological factors in NSCLC

The relationship between the clinicopathological factors of NSCLC patients with wild-type *TP53* and PICT1 expression are shown in Table 1. Nucleolar and cytoplasmic PICT1 expression were determined to be positive or negative. There was no correlation between nucleolar PICT1 expression and clinicopathological factors. However, cytoplasmic PICT1 expression was significantly correlated with sex (*P* = 0.013), smoking history (*P* = 0.005), differentiation (*P* = 0.001), lymphatic invasion (*P* = 0.012) and pathological stage (*P* = 0.012). Lung cancer tissue from males, smokers, moderate or poor differentiation, positive lymphatic invasion and advanced stage showed significantly higher positive rates of cytoplasmic PICT1 expression. In the histological subgroup analysis, squamous cell carcinoma tissue had a tendency to show higher positive rates of both nucleolar and cytoplasmic PICT1 expression than adenocarcinoma tissues (Figure 1D and Table 1), although this was not statistically significant.

**Figure 2:**
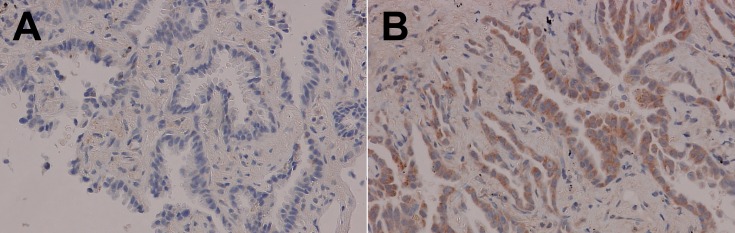
Cytoplasmic PICT1 expression in adenocarcinoma with lymphatic invasion Lung cancer tissue with lymphatic invasion had a significantly higher rate of positive cytoplasmic PICT1 expression than samples without lymphatic invasion. (A) Negative cytoplasmic PICT1 in adenocarcinoma with negative lymphatic invasion (200×). (B) Positive cytoplasmic PICT1 staining in adenocarcinoma with positive lymphatic invasion (200×).

Multivariate analysis using a logistic regression model (Table 3) with factors proven to be significant in the chi-square analysis revealed that only lymphatic invasion was significantly associated with cytoplasmic PICT1 expression (hazard ratio: HR 5.02, 95% C.I. 1.20– 27.51, *P* = 0.026). Lung cancer tissue (wild-type *TP53*) with positive lymphatic invasion had a significantly higher positive rate of cytoplasmic PICT1 expression (Figure 2).

**Table 3:** Multivariate analysis of the association between clinicopathological factors and cytoplasmic PICT1 expression

	PICT1 expression in cytoplasm		
Factors	Hazard ratio	95% CI	*P* value
Sex (male/female)	0.45	0.14 - 1.37	0.159
Smoking history	1.96	0.64 - 6.30	0.240
Differentiation (well vs moderate/poor)	2.63	0.99 - 7.15	0.053
Lymphatic invasion	5.02	1.20 - 27.51	0.026*
Pathological stage (I+II vs III+IV)	5.82	0.85 - 118.4	0.077

* *P* < 0.05

Abbreviation: CI, confidence interval

## DISCUSSION

This study scrutinized the expression of PICT1 in the nucleoli and cytoplasm of NSCLC cells with wild-type *TP53*. PICT1 has been known to be a nucleolar protein; however, in our study many NSCLC tissues expressed PICT1 not only in the nucleoli but also in the cytoplasm. Although there was no correlation between nucleolar PICT1 expression and clinicopathological factors in NSCLC patients with wild-type *TP53*, cytoplasmic PICT1 expression was significantly associated with several clinicopathological factors (male, smoker, moderate or poor differentiation, advanced stage and lymphatic invasion) in these patients. It is well known that the mutation rate of *TP53* is particularly high in current smokers [13-15]. In this study, positive cytoplasmic PICT1 expression was associated with smoking in NSCLC patients with wild-type *TP53*. Other factors, such as moderate or poor differentiation and advanced stage, were considerably poor prognostic factors. Multivariate analysis revealed that lymphatic invasion was the sole factor associated with cytoplasmic PICT1 expression; lymphatic vessel invasion has previously been considered an independent poor prognostic factor in surgically managed NSCLC [16-19]. Furthermore, *TP53* mutation or overexpression was demonstrated to be an indicator of poor prognosis in NSCLC [15, 20, 21]. Although the role of PICT1 in lymphatic invasion by tumor cells is not known, our results suggested the possibility that cytoplasmic PICT1 is associated with malignant behavior in NSCLC patients with wild-type *TP53*.

Although the expression of PICT1 in some tumors has been investigated immunohistochemically, it is controversial whether PICT1 is associated with malignant potential in cancer cells [8, 22]. PICT1 was previously considered a suppressor of tumor development [8-10], and low PICT1 expression in astrocytic glial tumors and ovarian cancers was correlated with high malignant progression [8, 9, 12]. This is the first report of PICT1 expression in NSCLC. We previously reported that PICT1 was a potential regulator of the MDM2–TP53 pathway and promoted tumor progression [4]. In fact, low PICT1 mRNA expression was associated with better prognosis in colorectal tumors, esophageal tumors, hepatocellular carcinomas and gastric cancers with wild-type *TP53*, therefore PICT1 was considered to be a useful prognostic marker for these cancers [4, 6, 7]. Together with our previous report and current study, we speculate that PICT1 expression might be associated with the malignant potential and tumor progression of NSCLC.

We previously reported the shorter survival in high PICT1 mRNA level patients with several cancers, and PICT1 thought to be a key regulator of tumor progression, [4, 6, 7]. However, the relationship between PICT1 protein localization and clinicopathological factors in patients with cancers has not been known. So, this is also the first report about the association between PICT1 protein expression and clinicopathological factors in patients with cancers. We reported that there were no significant differences between high and low PICT1 expression in several clinichopathological factors of hepatocellular carcinoma patients with wild type *TP 53*[6]. In gastric cancer patients with wild-type *TP53*, PICT1 expression was significantly associated with tumor depth[7]. There were no significant association between lymphatic invasion and PICT1 level in these patients[7]. In NSCLC with wild type *TP53*, we found that cytoplasmic PICT1 expression was significantly associated with several clinichopathological factors.

PICT1 binds to RPL11 in the nucleolus and prevents it from interacting with MDM2, thus blocking TP53 accumulation and activation [4]. Upon nucleolar stress, ribosomal protein RPL5, RPL11, RP23 and RPS7 is released from the nucleolus, binds MDM2 and activates TP53 [23-27]. We previously reported that although most of the PICT1 located in nucleolus, some of them also located in cytoplasm[4]. In this study, cytoplasmic PICT1 expression was correlated with several clinicopathological factors, but the role of this subcellular localization pattern is not yet known. Bursac et al. reported recently that upon exposure of cells to various ribosomal stressors, RPL5 and RPL11 accumulate in the ribosome-free fraction where they specially bind MDM2 [28]. A significant increase in the amount of ribosome-free RPL11 in the cytoplasm was observed, and TP53 activation might be required in response to ribosomal biogenesis stressors [28]. Another ribosomal protein, RPS27L regulate p53 function. RPS27L is mainly localized in cytoplasm under unstressed condition, but RPS27L is shuttled to nucleus, upon DNA damage and ribosomal stress, where it co-localized with MDM2[29]. Although further study will be required to address the significance of cytoplasmic PICT1 protein expression, together with results of this study, these predictions raise the possibility that PICT1 in the cytoplasm might also be important in tumor progression to control TP53 accumulation by regulating localization of ribosomal protein in the cytosolic ribosomal fraction, as well as nucleolar PICT1 to retain RPL11 in the nucleolas.

In conclusion, we scrutinized PICT1 expression in samples of NSCLC with wild-type *TP53*, and showed a potential correlation between cytoplasmic PICT1 expression and several clinicopathological factors in these patients. The results indicated that cytoplasmic PICT1 expression was a significant poor prognostic factor in NSCLC. Our results suggest the possibility that PICT1 promotes tumor progression in NSCLC via lymphatic invasion. Further studies are required to identify the precise role of PICT1 in lung cancer patients. Moreover, *TP53* status may be of great value in the choice of chemoradiation therapy, and the TP53 pathway might be a therapeutic target [15]. When this is fully determined, we will have a novel approach with which we can explore personalized and targeted therapy in PICT1-positive lung cancer patients with wild-type *TP53*.

## MATERIALS AND METHODS

### Patients and sample collection

In this retrospective study, we analyzed specimens from 96 NSCLC patients with wild-type *TP53* (86 adenocarcinoma, 10 squamous cell carcinoma) who had undergone surgical resection for lung cancer at the Department of Surgery and Science, Kyusyu University Hospital in Japan, from January 2009 to March 2011.

Surgical specimens were fixed in neutral-buffered formaldehyde, and processed for histopathological and immunohistochemical evaluation. Histological subtype of tumors and pathological stage were classified according to the WHO 2004 classification [30] and UICC guidelines of TNM classification (version 7), respectively [31]. This study was approved by the Ethics Committee of Kyusyu University.

### Immunohistochemistry

The Human Protein Atlas site (the project is funded by the Knut & Alice Wallenberg foundation) detailed the results of PICT1 expression in several malignant tissues. They showed that PICT1 expression was positive in both the cytoplasm and nuclei of lung cancer tissues.

Paraffin sections of surgically resected specimens were routinely deparaffinized and rehydrated [32]. The sections were incubated overnight at 4°C with primary rabbit polyclonal antibody against PICT1, GLTSCR2 (PICT1: HPA018999, Sigma–Aldrich), then incubated with secondary antibody conjugated with streptavidin-biotin peroxidase (Histofine SAB-PO kit, Nichirei, Tokyo, Japan), and visualized with 3,3′-diaminobenzidine (DAB). Normal brain section was used as a positive control for PICT1 [8]. Parallel negative controls omitting primary antibody were also prepared, and did not show background (data not shown). All the immunoreactions were separately evaluated by two investigators (K.O. and K.T.) without prior knowledge of patients' clinical records. Nucleolar and cytoplasmic staining were evaluated separately. Tumor cells with brown staining in the nucleolus or cytoplasm were regarded as positive *cf.* Figure 1.

### Mutation spectrum analysis for *TP53* gene

We immediately froze the resected lung cancer tissues in liquid nitrogen. Genomic DNA was extracted from tissue samples. We determined mutational status of *TP53* by sequencing a DNA region spanning exons 5 to 9, the area where most *TP53* mutations occur as reported previously [33, 34]. Briefly, the DNA fragments were amplified by polymerase chain reaction (PCR) using each primer pair. Mutations in *TP53* were detected by PCR direct sequencing of all PCR products with the ABI Prism 310 Genetic Analyzer (Applied Biosystems).

Then we chose all lung cancer tissues without *TP53* mutation as being wild-type for *TP53*, and examined PICT1 expression.

### Statistical analysis

Average scores were expressed as mean ± standard deviation (SD). Chi-square tests were used to analyze the correlation between clinicopathological factors and PICT1 immunoreactivity. Logistic regression model was used for the multivariate analysis of the association between PICT1 expression and clinicopathological factors proven to be significant in the chi-square test. *P* values less than 0.05 were considered to be statistically significant. JMP version 9 (SAS Institute, Inc., Cary, NC, USA) software was used for all analyses.
